# Intramuscular injection of adenoviral hepatocyte growth factor at a distal site ameliorates dextran sodium sulfate-induced colitis in mice

**DOI:** 10.3892/ijmm.2014.1686

**Published:** 2014-03-06

**Authors:** KENTARO YUGE, TOMOYUKI TAKAHASHI, NGIN CIN KHAI, KAZUKO GOTO, TAKAKO FUJIWARA, HISAYOSHI FUJIWARA, KEN-ICHIRO KOSAI

**Affiliations:** 1 Department of Gene Therapy and Regenerative Medicine, Graduate School of Medicine, Gifu University, Gifu 502-1194, Japan; 2 Division of Respirology, Neurology and Rheumatology, Department of Medicine, Kurume University School of Medicine, Kurume 830-0011, Japan; 3 Division of Gene Therapy and Regenerative Medicine, Cognitive and Molecular Research Institute of Brain Diseases, Kurume University, Kurume 830-0011, Japan; 4 Department of Gene Therapy and Regenerative Medicine, Kagoshima University Graduate School of Medical and Dental Sciences, Kagoshima 890-8544, Japan; 5 Department of Cardiology, Graduate School of Medicine, Gifu University, Gifu 502-1194, Japan; 6 Department of Food Science, Kyoto Women’s University, Kyoto 605-8501, Japan; 7 Department of Cardiology, Hyogo Prefectural Amagasaki Hospital, Amagasaki, Hyogo 660-0828, Japan

**Keywords:** hepatocyte growth factor, inflammatory bowel disease, gene therapy, adenoviral vector, antiapoptosis, immunomodulation, regeneration, cytokine, clinical application

## Abstract

Inflammatory bowel disease (IBD) severely affects the quality of life of patients. At present, there is no clinical solution for this condition; therefore, there is a need for innovative therapies for IBD. Hepatocyte growth factor (HGF) exerts various biological activities in various organs. However, a clinically applicable and effective HGF-based therapy for IBD has yet to be developed. In this study, we examined the therapeutic effect of injecting an adenoviral vector encoding the human HGF gene (Ad.HGF) into the hindlimbs of mice with dextran sodium sulfate (DSS)-induced colitis. Plasma levels of circulating human HGF (hHGF) were measured in injected mice. The results showed that weight loss and colon shortening were significantly lower in Ad.HGF-infected mice as compared to control (Ad.LacZ-infected) colitic mice. Additionally, inflammation and crypt scores were significantly reduced in the entire length of the colon, particularly in the distal section. This therapeutic effect was associated with increased cell proliferation and an antiapoptotic effect, as well as a reduction in the number of CD4^+^ cells and a decreased CD4/CD8 ratio. The levels of inflammatory, as well as Th1 and Th2 cytokines were higher in Ad.HGF-infected mice as compared to the control colitic mice. Thus, systemically circulating hHGF protein, produced by an adenovirally transduced hHGF gene introduced at distal sites in the limbs, significantly ameliorated DSS-induced colitis by promoting cell proliferation (i.e., regeneration), preventing apoptosis, and immunomodulation. Owing to its clinical feasibility and potent therapeutic effects, this method may be developed into a clinical therapy for treating IBD.

## Introduction

The breakdown of normal mucosal immunity causes the development of inflammatory bowel disease (IBD), which can be classified as Crohn’s disease (CD) and ulcerative colitis (UC) (1). IBD is a chronically relapsing and remitting condition of unknown origin that exhibits various features of immunological inflammation and affects at least 1 in 1,000 people in western countries. IBD is characterized by inflammation in the intestine, and is associated with diarrhea, occult blood, abdominal pain, weight loss, anemia and leukocytosis. IBD primarily affects young adults, and the disease initially manifests in childhood in 15–25% of cases. Therefore, IBD patients often develop severe symptoms that decrease their quality of life (2). Consequently, there is a need for innovative therapies for IBD.

Current treatments for IBD focus on suppressing inflammation or modulating the immune response using corticosteroids, mercaptopurines, 5-ASA, or monoclonal antibodies against inflammatory cytokines, e.g., the anti-tumor necrosis factor (TNF)-α antibody infliximab (3). However, despite the wide variety of pharmacologic options for patients with IBD, consistent cures and prolonged remissions have yet to be achieved.

Hepatocyte growth factor (HGF) was originally identified (4–7) and cloned (8,9) as a potent mitogen for hepatocytes, but has since been established as a multifunctional cytokine that exhibits mitogenic, motogenic, morphologic, angiogenic, antiapoptotic and organotrophic effects in a variety of tissues (10). HGF is upregulated in inflamed colonic mucosal tissue in patients with CD or UC (11–13), and plasma HGF levels are elevated in animal models of acute colitis (14). *In vitro*, HGF modulates intestinal epithelial cell proliferation and migration (15), thereby enhancing epithelial cell restitution, which is the initial step of gastrointestinal wound healing. In addition, administration of recombinant human HGF (hHGF) protein reduces the severity of colitis and accelerates colonic mucosal repair in models of TNBS-induced and DSS-induced colitis (16–19), as well as in HLA-B27 transgenic rats with colitis (20). Mukoyama *et al* (21) showed that the intrarectal administration of an adenoviral (Ad) vector carrying the HGF gene prevented TNBS-induced colitis. Additionally, Hanawa *et al* (22) demonstrated the attenuation of mouse DSS colitis by naked gene transfer of rat HGF into the liver, and Kanbe *et al* (23) reported the amelioration of mucosal damage in DSS colitis by the intrarectal administration of the naked HGF gene. In their study, Kanayama *et al* (24) demonstrated the promotion of colonic epithelial regeneration by HGF gene transfer through electroporation. Findings by those authors suggest that HGF is potentially an important new treatment modality for promoting the repair of intestinal mucosa in patients with IBD.

In the majority of previous studies, HGF was provided in the form of recombinant hHGF protein. However, due to the rapid clearance of the HGF protein, large doses and frequent administration of recombinant hHGF were required. Naked gene transfer is a simple and easy method, but the efficiency of gene transduction is extremely low, possibly leading to insufficient clinical effectiveness in human patients. By contrast, the intrarectal administration of an Ad carrying the HGF gene is considered to be extremely stressful for patients. Therefore, in this study we injected an Ad carrying the hHGF gene in single rounds of injections into both hindlimbs of mice 1 day after administration of DSS. We then investigated the therapeutic effects and mechanisms of systemically circulating HGF protein, produced by a gene introduced into the limbs, in the DSS-induced acute colitis model.

## Materials and methods

### Recombinant Ad

The Ad expressing hHGF under the transcriptional control of the cytomegalovirus immediate-early enhancer and a modified chicken β-actin promoter (Ad.HGF) was generated as described previously (25). The Ad.HGF and the control Ad expressing the LacZ gene (Ad.LacZ) were amplified in HEK-293 cells, purified twice on CsCl gradients, and desalted as described previously (26–29).

### Animal studies

Six- to 7-week-old female BALB/c mice weighing 17–20 g (Japan SLC, Inc., Hamamatsu, Japan) were housed in cages in a temperature-controlled environment under a 12-h light-dark cycle with free access to food and water. The animal studies were performed in accordance with the National Institutes of Health guidelines, as specified by the Animal Care Facility at Gifu University School of Medicine.

To induce dextran sodium sulfate (DSS) colitis, the mice were provided with distilled drinking water containing 5% (w/v) DSS (MW, 36,000–50,000; ICN Biomedicals Inc., Aurora, OH, USA) for 7 days. Subsequently, colitis was maintained by feeding the mice 1% DSS (30–32) in the drinking water.

One day after the administration of DSS, Ad.HGF was injected into both hindlimbs of each mouse for a total dose of 1×10^11^ particles/mouse (i.e., 5×10^10^ particles each into the left and right thigh muscles) (n=8). Ad.LacZ was injected in a similar manner into control mice (n=8). These groups were followed until day 15 (i.e., 8 days after the end of the 7-day period of 5% DSS administration). To evaluate the severity of colitis, body weight was examined on a daily basis. On day 15, all the mice were sacrificed by inhaled anesthetics, and colon samples were collected for examination. In other experiments, on day 5 of 5% DSS administration, 5-bromo-2′-deoxyuridine (BrdU, 100 mg/kg) was administered intraperitoneally to mice (n=8) infected with Ad.HGF or Ad.LacZ, and the animals were sacrificed by inhaled anesthetics 2 h later. These samples were used for analyses of HGF signal transduction, cell proliferation, apoptosis, cytokines and lymphocyte surface markers. The concentration of exogenous hHGF in serum was analyzed using the same dose (i.e., 1×10^11^ particles/mouse) of Ad.LacZ or Ad.HGF in intact mice (n=16).

### Enzyme-linked immunosorbent assay

The plasma concentration of hHGF following adenoviral intramuscular gene transduction (IMGT) was measured in mice at each time point (n=4) using the Quantikine human HGF Immunoassay kit (R&D Systems, Inc., Minneapolis, MN, USA). TNF-α, interleukin (IL)-1β, IL-6, interferon (IFN)-γ, IL-2, IL-4 and IL-5 levels in the colons of colitic mice were measured using commercially available enzyme-linked immunosorbent assay (ELISA) kits (BioSource International, Inc., Camarillo, CA, USA) according to the manufacturer’s instructions.

### Immunoprecipitation and c-Met receptor phosphorylation assay

The phosphorylation and activation of the c-Met receptor in colon tissues were detected by immunoprecipitation, as described previously (33,34). In brief, 1 g of colon tissue was homogenized in 4 ml of lysis buffer [1% Triton X-100, 150 mM NaCl, 50 mM Tris-HCl (pH 7.6), 10% glycerol, 1 mM vanadate, and 1 mM phenylmethylsulfonyl fluoride] with a protease-inhibitor cocktail (Sigma-Aldrich, Tokyo, Japan). Following centrifugation, the supernatant was incubated with 0.5 μg/ml anti-mouse c-Met antibody (sc-162; Santa Cruz Biotechnology, Inc., Dallas, TX, USA) for 4 h, and then sequentially incubated with 5 μl of protein G-Sepharose beads for 3 h. After washing, proteins bound to the beads were dissolved in sample buffer and subjected to SDS-PAGE. Phosphorylated c-Met was immunoblotted using the anti-phosphotyrosine antibody PY20 (Transduction Laboratories, Lexington, KY, USA).

### Histopathological analysis

After each mouse was sacrificed, the intestine was dissected from the anus to the cecum and rinsed with physiological saline. The colon length was measured, and the colon sample was divided into three sections (cecum, proximal colon and distal colon), with the cecum being separated first, and then the remaining part of the colon being divided into two equal segments (proximal colon and distal colon). The cecum, proximal colon and distal colon were opened longitudinally, and the proximal and distal colon were equally divided longitudinally and transversely. Thus, the cecum was divided into two sections, and the proximal and distal colon were divided into four sections. The colon tissues were fixed in 10% formalin and embedded in paraffin, and 4-μm sections were cut and stained with hematoxylin and eosin (H&E) to determine the inflammation and crypt scores (35). Briefly, the sections were graded on a scale of 0–3 to indicate the severity of inflammation: 0, none; 1, mucosa; 2, mucosa and submucosa; and 3, transverse, and on a scale of 0–4 to indicate the severity of crypt damage: 0, none; 1, basal 1/3 damage; 2, basal 2/3 damage; 3: loss of the entire crypt with the surface epithelium remaining intact; and 4, loss of the entire crypt and surface epithelium. The changes were also scored with regard to the extent of tissue involvement, measured as a percentage: i) 1–25%, ii) 26–50%, iii) 51–75%, and iv) 76–100%. Each section was then separately scored for each feature by taking the product of the severity score and the score for the extent of tissue involvement. Thus, the inflammation score ranged from 0 to 12, and the crypt score ranged from 0 to 16. Apoptotic cells were detected using a light microscope (Olympus, Tokyo, Japan) and the terminal deoxynucleotidyltransferase-mediated deoxyuridine triphosphate biotin nick end-labeling (TUNEL) assay (ApopTag kit; Intergen Co., Purchase, NY, USA), as described previously (25,33,36). To detect proliferating cells, BrdU incorporation was measured using a staining kit (Zymed Laboratories, Inc., South San Francisco, CA, USA) according to the manufacturer’s instructions.

Endothelial cells, CD4^+^ T lymphocytes, and CD8^+^ T lymphocytes were detected *in situ* using an anti-vWF antibody (Dako Cytomation Co., Ltd., Kyoto, Japan), anti-CD4 antibody and anti-CD8 antibody (both from Zymed Laboratories, Inc.), respectively, as described previously (25,36).

### Statistical analysis

Values provided are the means ± SEM values. The significance of differences was evaluated using the Student’s t-test.

## Results

### Intramuscular injection of Ad.HGF produces circulating plasma hHGF, leading to c-Met activation in the colonic mucosa

DSS-induced colitis was induced in 6- to 7-week-old female BALB/c mice. One day after DSS administration, Ad.HGF was administered in a single procedure involving injections into both hindlimbs (total dose, 1×10^11^ particles/mouse; as mentioned in Materials and methods). In the hHGF-overexpressing mice, the plasma levels of hHGF were 1,140±101, 634±341 and 33.9±15.8 pg/ml at 2, 4 and 6 days after injection, respectively. No hHGF was detected in the Ad.LacZ-treated mice at any time point, demonstrating that this method accurately detected only hHGF protein expressed from the hHGF transgene, without a cross-reaction resulting in detection of the endogenous mouse HGF protein. These results indicate that hHGF expression was effectively induced by the intramuscular injection of Ad.HGF, leading to the presence of hHGF in the plasma of the mice.

**Figure 1:**
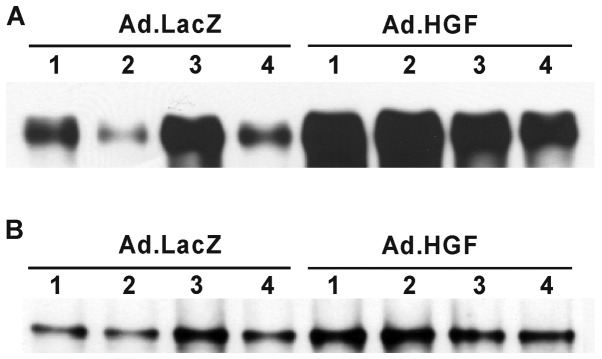
Tyrosine phosphorylation of c-Met in the colon epithelium. Colonic mucosal tissue of dextran sodium sulfate (DSS)-treated mice injected with Ad.LacZ (n=4) or Ad.HGF (n=4) was solubilized in lysis buffer. Lysates were immunoprecipitated with anti-c-Met antibody and blotted with (A) anti-phosphotyrosine antibody or (B) anti-c-Met antibody. Each lane represents the colonic tissue lysate of individual animals. Adenoviral human hepatocyte growth factor (hHGF) intramuscular gene transduction (IMGT) led to the strong stimulation of c-Met phosphorylation in colonic mucosal tissue.

The biological effects of HGF are mediated by its receptor c-Met, which is capable of activating multiple intracellular transducers and signaling pathways. Therefore, we examined c-Met tyrosine phosphorylation in the colonic mucosal epithelium by western blotting (Fig. 1). Phosphorylated c-Met was detected at low or moderate levels in the injured colonic mucosa of mice treated with Ad.LacZ, presumably as a result of a DSS-induced increase in endogenous HGF in response to colonic mucosal injury (14). By contrast, the injured colonic mucosa of mice treated with Ad.HGF exhibited high levels of c-Met tyrosine phosphorylation.

### Adenoviral hHGF IMGT prevents weight loss in DSS-induced colitis mice

**Figure 2:**
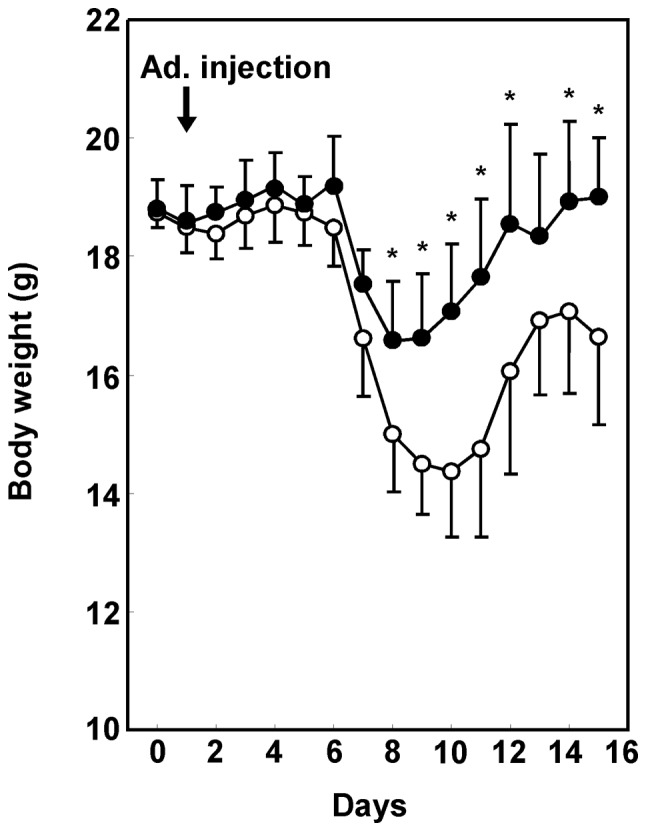
Adenoviral human hepatocyte growth factor (hHGF) intramuscular gene transduction (IMGT) ameliorated weight loss. Mice were given distilled drinking water containing 5% dextran sodium sulfate (DSS) for 7 days and 1% DSS for 8 days, *ad libitum*. One day after DSS administration, Ad.HGF (closed circles; n=8) was injected into both hindlimb muscles of 8 mice. As a control, Ad.LacZ (open circles; n=8) was injected into both hindlimb muscles of another group of 8 mice. Ad.HGF injection significantly prevented weight loss in colitic mice. ^*^P<0.05.

DSS-induced colitis is characterized by bloody stools and severe weight loss (30). In mice treated with Ad.LacZ, we observed persistent liquid stool and waste with subsequent severe weight loss. By contrast, colitic mice that received a single round of injections of Ad.HGF exhibited significant reductions in liquid stool and gross bleeding from the rectum (data not shown). Fig. 2 shows the mean weight change, and that the body weights of Ad.HGF-treated mice were significantly higher than those of the Ad.LacZ-treated mice. In the Ad.LacZ-treated control mice, weight loss occurred 6–7 days after the initiation of DSS administration. Ad.HGF treatment significantly prevented this weight loss.

### Adenoviral hHGF IMGT reduces colitis-induced intestinal shortening and pathological scores

**Figure 3:**
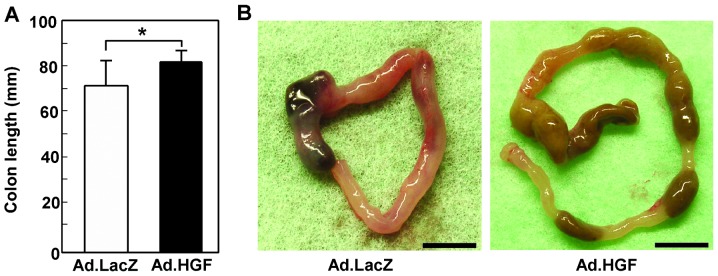
Adenoviral human hepatocyte growth factor (hHGF) intramuscular gene transduction (IMGT) reduced inflammation in the colon and prevented colon shortening in dextran sodium sulfate (DSS)-induced colitis. Colon lengths were measured from the colocecal junction to the anal verge on day 15 (Ad.LacZ, n=8; Ad.HGF, n=8). (A) Ad.HGF treatment prevented shortening of the colon in mice with DSS-induced colitis. ^*^P<0.05. Representative colon pictures from the Ad.LacZ- and Ad.HGF-injected groups are shown in (B). The scale bar indicates 1 cm.

Shortening of the colon correlates well with histologic changes, and colon length is therefore frequently used as a morphologic parameter to indicate the degree of inflammation (35). The colon lengths of mice treated with Ad.LacZ and Ad.HGF were 72.0±10.6 and 82.0±4.7 mm, respectively (Fig. 3A). In contrast to the colons in the Ad.HGF-treated group, the colons in the Ad.LacZ-treated group were short and severely inflamed, with evident hemorrhages (Fig. 3B).

**Figure 4:**
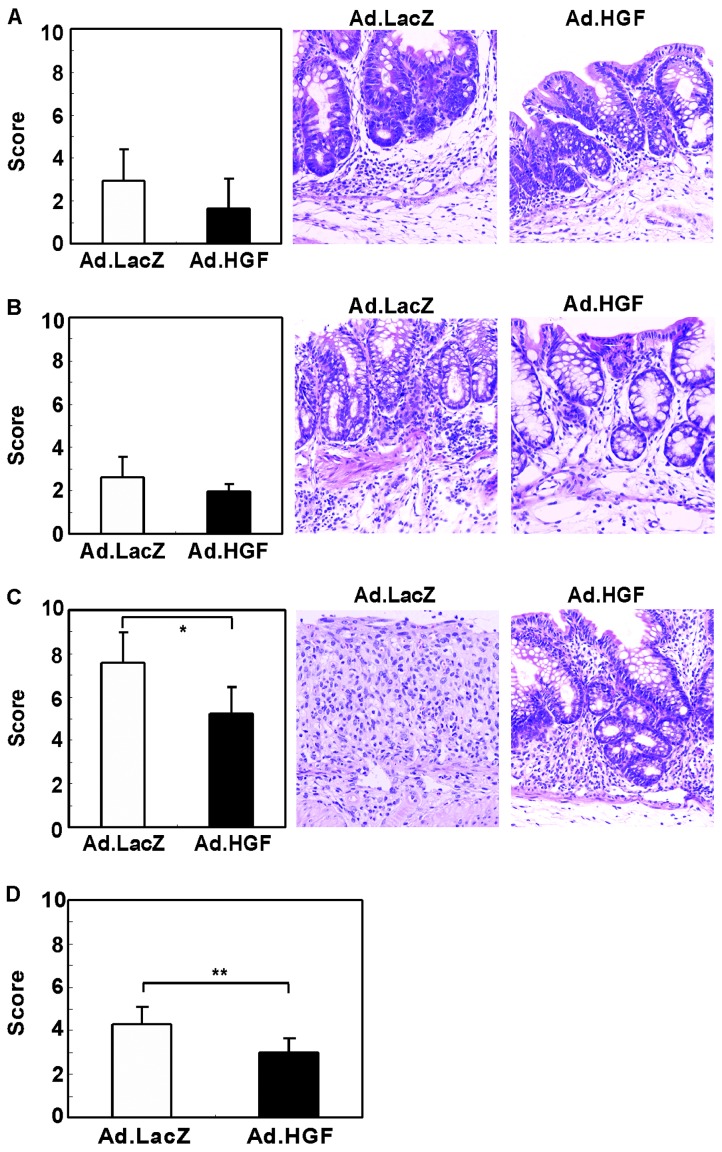
Adenoviral human hepatocyte growth factor (hHGF) intramuscular gene transduction (IMGT) decreased colon inflammation in dextran sodium sulfate (DSS)-induced colitis. (A) Cecum, (B) proximal, (C) distal, and (D) total colon samples from the anal ring were used for histological evaluation. Colonic tissues taken on day 15 were stained with hematoxylin and eosin (representative histopathological images are shown on the right) (original magnification, ×100). Histological scoring of the severity of inflammation was performed in a blind manner (graph on the left). Infiltration of inflammatory cells was significantly reduced in the adenoviral HGF treatment group. ^*^P<0.05 and ^**^P<0.01.

**Figure 5:**
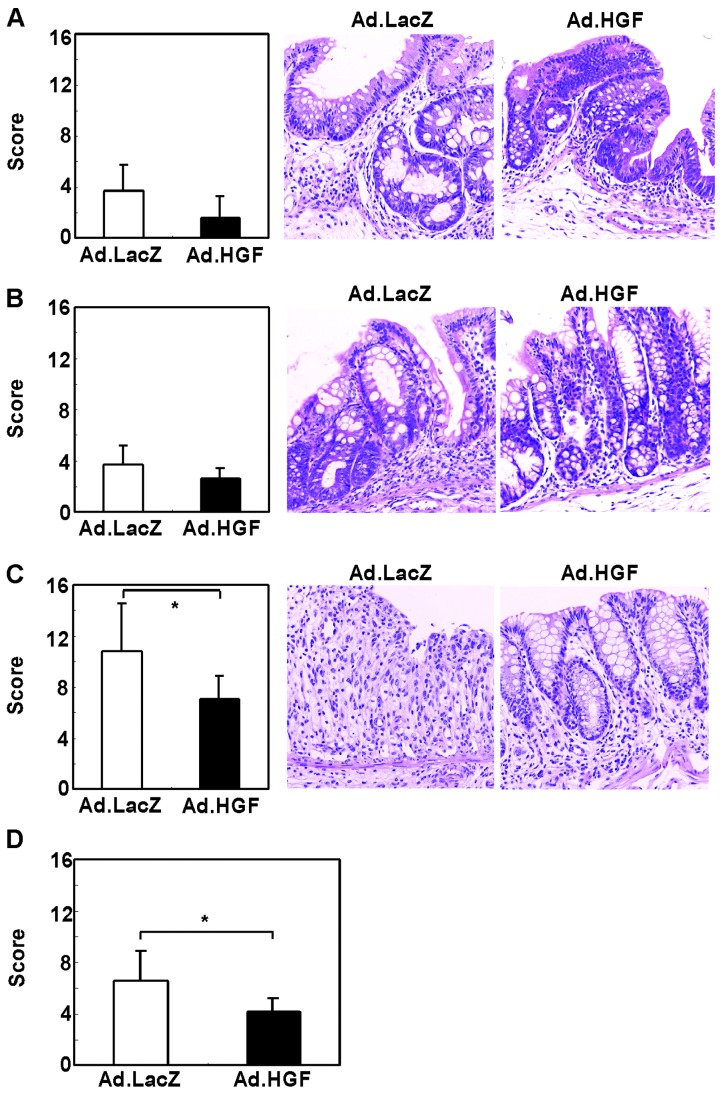
Adenoviral human hepatocyte growth factor (hHGF) intramuscular gene transduction (IMGT) prevented crypt destruction in dextran sodium sulfate (DSS)-induced colitis. (A) Cecum, (B) proximal, (C) distal, and (D) total colon samples from the anal ring were used for histological evaluation. Colonic tissues taken on day 15 were stained with hematoxylin and eosin (representative histopathological images are shown on the right; original magnification, ×100). Histological scoring of the severity of crypt damage was performed in a blind manner (graph on the left). Crypt damage was significantly reduced in the adenoviral hHGF treatment group. ^*^P<0.05.

To validate this finding, we evaluated the effect of Ad.HGF on DSS-induced colonic mucosal injury in mice by histological analysis at day 15. In the cecum and proximal part of the colon (i.e., towards the end of the cecum), the inflammation and crypt scores appeared to be decreased by Ad.HGF administration although this difference was not statistically significant (Figs. 4A and B, 5A and B). By contrast, treatment with Ad.HGF significantly decreased the inflammation and crypt scores in the distal part (i.e., towards the anus) and in the colon overall (Figs. 4C and D, 5C and D).

### Kinetics of inflammation in colitic mice

**Figure 6:**
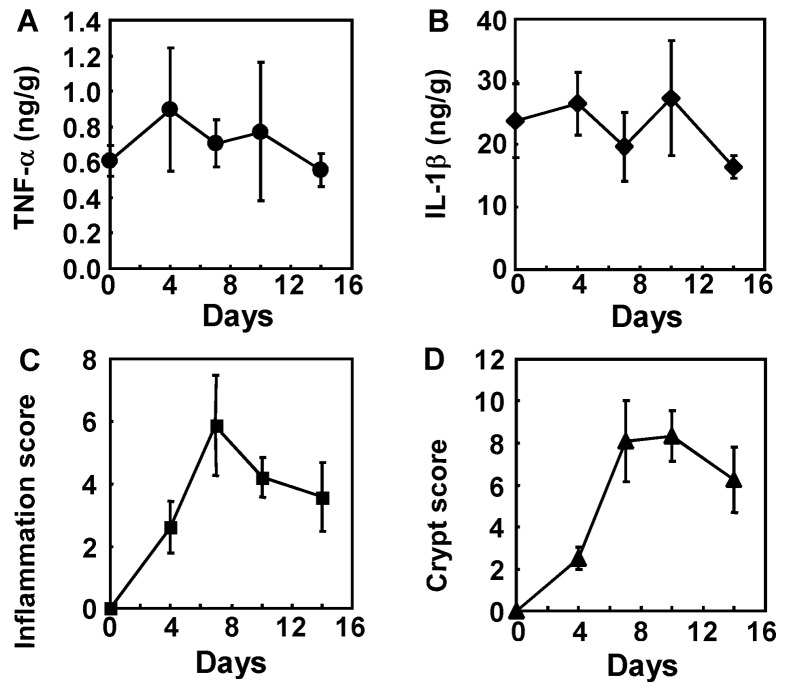
Expression of tumor necrosis factor (TNF)-α and interleukin (IL)-1β, and inflammation and crypt scores, in dextran sodium sulfate (DSS)-induced colitis. Twenty mice were given distilled drinking water containing 5% DSS for 7 days and 1% DSS for 7 days, *ad libitum*. Five mice were sacrificed at days 4, 7, 10 and 14. Analyses were performed to determine (A) TNF-α and (B) IL-1β expression in the colon per gram of total colon tissue, (C) inflammation score, and (D) crypt score. TNF-α and IL-1β expression increased on days 4 and 10, the inflammation score peaked at day 7, and the crypt score peaked at days 7 and 10.

To elucidate the mechanism underlying the therapeutic effect of hHGF, we studied the expression of TNF-α and IL-1β in the colon and evaluated the inflammation and crypt scores at days 4, 7, 10 and 14 of the experimental colitis model (Fig. 6). The expression of TNF-α and IL-1β peaked as early as day 4 (Fig. 6A and B). The inflammation and crypt scores peaked as early as day 7 (Fig. 6C and D). Given that the plasma concentration of hHGF protein peaked on day 2 and decreased thereafter, colon tissue were sampled and hHGF functions were analyzed on day 5.

### Adenoviral hHGF IMGT suppresses apoptosis and enhances regeneration of the colonic epithelium

**Figure 7:**
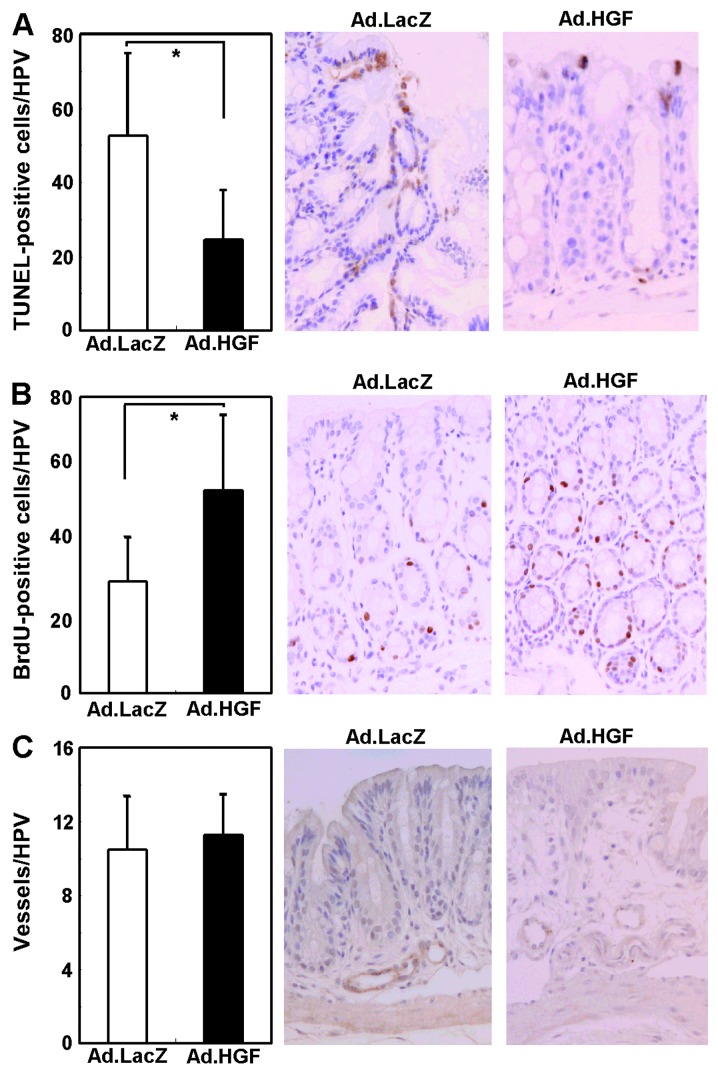
Adenoviral human hepatocyte growth factor (hHGF) intramuscular gene transduction (IMGT) prevented apoptosis and stimulated intestinal epithelial regeneration in dextran sodium sulfate (DSS)-induced colitis. Colon tissues were stained by immunohistochemistry (representative histopathological images are shown on the right) (original magnification, ×100). The graphs indicate the average number of positive cells or vessels per high-power field (left column). (A) TUNEL staining of the distal colon from Ad.LacZ-treated and Ad.HGF-treated mice. The graph indicates the number of apoptotic cells detected in the epithelial crypts. A single round of Ad.HGF injection into both hindlimbs almost completely prevented apoptosis in the colon epithelium. (B) 5-Bromo-2′-deoxyuridine (BrdU) staining of the distal colon from Ad.LacZ-treated and Ad.HGF-treated mice. In the Ad.HGF-treated mice, a significant increase in the amount of BrdU-incorporating cells was observed in the colon epithelium. (C) vWF staining of the distal colon from Ad.LacZ-treated and Ad.HGF-treated mice. No significant difference was observed in the number of vessels between the two groups. ^*^P<0.05.

In DSS-induced colitis, loss of colonic mucosal epithelial cells is closely associated with apoptosis (37,38). To evaluate the role of Ad.HGF in preventing apoptosis in colonic epithelial cells, we performed the TUNEL assay to detect apoptotic cells (Fig. 7A). Ad.HGF-treated colitic mice had significantly (2.1-fold) fewer TUNEL-positive cells per high-power field (HPF) than Ad.LacZ-treated colitic mice.

To determine whether Ad.HGF-injection stimulated the proliferation of colonic epithelial cells, we measured the DNA labeling index in the colonic mucosal epithelium. As shown in Fig. 7B, the average number of BrdU-positive cells in the colonic mucosal epithelium was significantly (1.8-fold) higher in Ad.HGF-treated as compared to Ad.LacZ-treated mice, suggesting that hHGF stimulates proliferation in the colonic epithelial cells of colitic mice. These results suggested that adenoviral hHGF IMGT promoted survival and regeneration of the colonic mucosal epithelium in mice with DSS-induced colitis. HGF is known to promote angiogenesis (10). Therefore, we hypothesized that the angiogenic effect of HGF may contribute to the repair of the damaged colonic epithelium. However, when we analyzed angiogenesis in the distal part of the colon by anti-vWF immunohistochemistry, the number of blood vessels in the colon did not differ significantly between Ad.HGF-treated mice and controls, although a few more vessels appeared to be present in Ad.HGF-treated animals (Fig. 7C).

### Effects of adenoviral hHGF IMGT on immunoreactive cells and inflammatory cytokines in DSS-induced colitis

**Figure 8:**
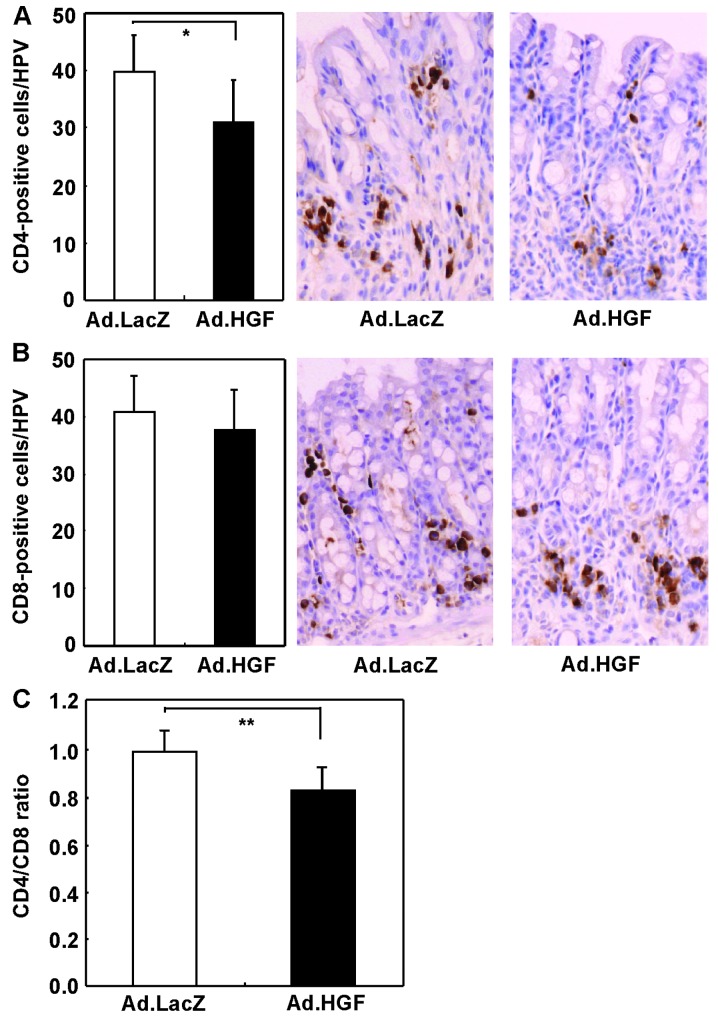
Effects of adenoviral human hepatocyte growth factor (hHGF) intramuscular gene transduction (IMGT) on inflammatory cells in dextran sodium sulfate (DSS)-induced colitis. (A) CD4 immunostaining of the distal colon. (B) CD8 immunostaining of the distal colon. The two images on the right are representative of immunostaining of CD4^+^ and CD8^+^ (original magnification, ×200), and the graphs on the left indicate the average number of positive cells per high-power field. (C) The graph indicates the CD4/CD8 ratio. The number of infiltrating CD4^+^ T cells and the CD4/CD8 ratio were decreased by adenoviral HGF IMGT. ^**^P<0.001 and ^*^P<0.05.

To determine whether IMGT of hHGF affected the immune system of DSS-treated mice, we directly detected immune cells in the colon. Adenoviral hHGF IMGT decreased the number of CD4^+^ T cells and the CD4/CD8 ratio, but not the number of CD8^+^ T cells (Fig. 8).

**Figure 9:**
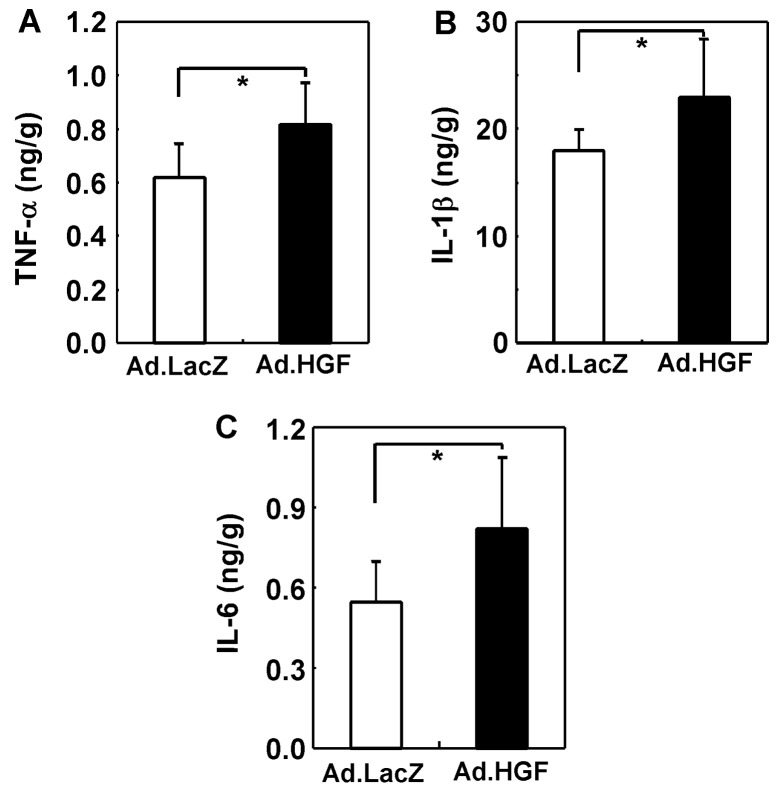
Effects of adenoviral human hepatocyte growth factor (hHGF) intramuscular gene transduction (IMGT) on inflammatory cytokines in dextran sodium sulfate (DSS)-induced colitis. On day 5 of DSS administration, the expression of inflammatory cytokines was evaluated by enzyme-linked immunosorbent assay (ELISA). The graphs indicate the level of each cytokine per gram of total colon tissue. The expression of inflammatory cytokines, (A) tumor necrosis factor (TNF)-α, (B) interleukin (IL)-1β, and (C) IL-6 increased after administration of adenoviral HGF IMGT. ^*^P<0.05.

The inflammatory cytokine cascade plays an important role in the pathogenesis of DSS-induced colitis. Therefore, we analyzed the cytokine profile of the entire colon by ELISA. In general, we observed upregulation of pro-inflammatory cytokines (TNF-α, IL-1β and IL-6) in the colitic mice (39,40). The expression levels of TNF-α, IL-1β and IL-6 were further increased by hHGF IMGT (Fig. 9).

**Figure 10:**
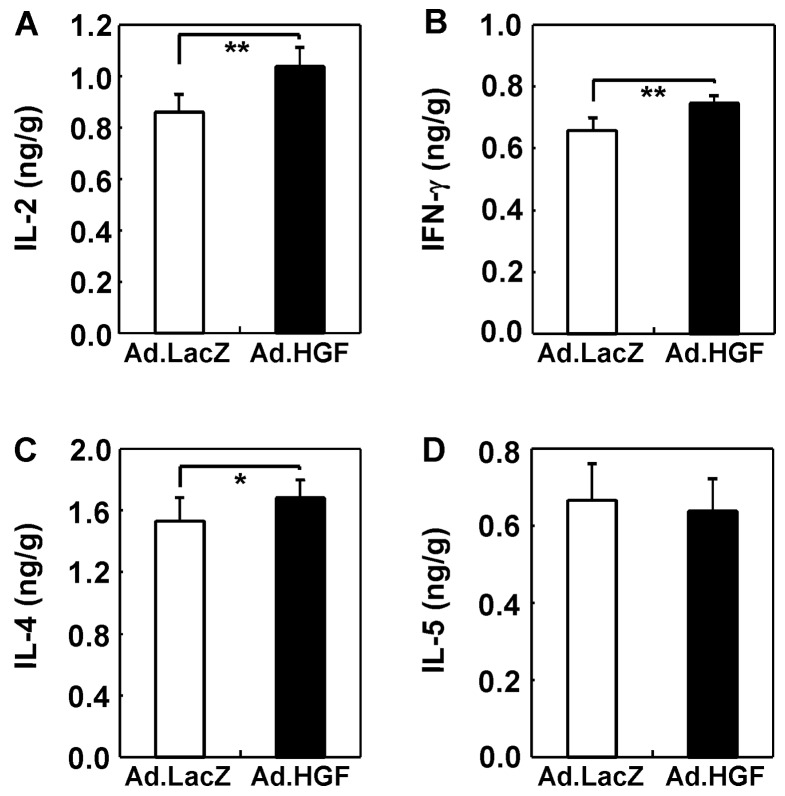
Effects of adenoviral human hepatocyte growth factor (hHGF) intramuscular gene transduction (IMGT) on Th1 and Th2 cytokines in dextran sodium sulfate (DSS)-induced colitis. The expression of the Th1 [(A) interleukin (IL)-2 and (B) interferon (IFN)-γ] and Th2 [(C) IL-4 and (D) IL-5] cytokines was determined by enzyme-linked immunosorbent assay (ELISA). The graphs indicate the expression of each cytokine per gram of total colon tissue. The expression of IL-2, IFN-γ and IL-4 increased after the administration of adenoviral HGF IMGT. ^*^P<0.05 and ^**^P<0.001.

We also examined the effect of hHGF IMGT on Th1 (IFN-γ and IL-2) and Th2 (IL-4 and IL-5) cytokine expression in the colons of colitic mice. IFN-γ, IL-2 and IL-4 were upregulated by hHGF treatment (Fig. 10).

## Discussion

This study evaluated the therapeutic potential of the intramuscular injection of HGF-expressing Ad for treating IBD, using a mouse model of DSS-induced colitis. The therapeutic strategy of adenoviral HGF IMGT, in which hHGF protein was produced at distal sites (hindlimbs) and systemically delivered to the target organ (the injured colon epithelium), functioned well. Epithelial cell injury in DSS-induced colitis was potently prevented by this method, which is clinically feasible, less invasive, and does not suffer from the drawbacks associated with the direct treatment of colitic tissues. Although previous studies (16–18) have shown that HGF exerts protective effects in bowel disease, the regimens tested involved high levels of recombinant HGF protein (>100 μg/kg) and repeated injections.

Recent advances in molecular techniques have provided several strategies for *in vivo* gene delivery, including naked plasmid DNA, liposomes encapsulating DNA, and viral vectors (41,42). For instance, Hanawa *et al* (22) reported that administration of the naked HGF gene into the liver attenuated acute colitis in mice, and Kanbe *et al* (23) showed that intrarectal administration of a plasmid carrying the HGF gene ameliorated DSS-induced colitis in mice. Kanayama *et al* (24) found that colonic epithelial regeneration is promoted by HGF gene transfer via electroporation. Oh *et al* (43) reported that HVJ liposomes encapsulating the hHGF gene ameliorated TNBS-induced colitis in mice, and that intrarectal administration of an Ad carrying the HGF gene improved colonic damage in TNBS-induced colitis (21). However, each type of gene therapy system used thus far has some associated limitations and concerns, particularly from the viewpoints of clinical applicability, feasibility and safety (41,42).

In this study, we assessed for the first time the therapeutic potential of a unique method of adenoviral hHGF IMGT for treating IBDs. In accordance with the results obtained in our previous studies of a mouse model of myocardial infarction (25,36), we successfully detected circulating hHGF in the plasma of colitic mice after adenoviral hHGF IMGT. In the colons of colitic mice that received adenoviral hHGF IMGT, the c-Met/HGF receptor was highly phosphorylated on tyrosine, demonstrating the functional efficacy of the adenoviral hHGF IMGT system. Furthermore, hHGF IMGT stimulated proliferation and inhibited apoptosis in the disrupted intestinal epithelial barrier. These results indicate that our hHGF IMGT system induces protection and regeneration in the colon, suggesting that it would be useful in clinical treatments for bowel diseases.

The effects of HGF on carcinogenesis remain unclear. Some studies suggest that HGF may promote the growth and metastasis of some cancer types, probably via the stimulation of cancer cell growth and angiogenesis (44,45). By contrast, carcinogenesis or malignant phenotypes in other cancer types are potently inhibited by overexpressed HGF (33). The effects of HGF on IBDs are also unclear. In general, tumor development may be caused by long-term exposure of cells to an abnormally overexpressed growth factor. In our therapeutic system, the duration of hHGF secretion after single rounds of intramuscular injection was relatively short; therefore, we consider the risk of cancer occurrence to be very low. In addition, a previous study demonstrated the efficacy of repeated administration of Ad into muscles, suggesting that this approach may yield sustained and elevated therapeutic efficiency: neutralizing antibodies against adenovirus should hinder only Ad circulating in the bloodstream, but not Ad administered into the muscle (46). These findings are encouraging with regard to the potential safety and clinical applicability of this approach.

With regard to the therapeutic mechanism, previous studies have reported that administration of recombinant HGF protein (16) and vector encoding HGF gene (43) ameliorate TNBS-induced colitis and reduced inflammation, decreasing the levels of inflammatory cytokines such as TNF-α. In particular, Oh *et al* (43) showed that administration of a plasmid carrying the HGF gene reduced the invasion of CD4^+^ cells and neutrophils and suppressed the expression of Th1 cytokines such as IL-12, IL-1β and IFN-γ in a TNBS-induced colitis model. Hanawa *et al* (22) showed that administration of an HGF gene-containing plasmid in the liver by intravenous injection suppressed the mRNA levels of IFN-γ, IL-18 and TNF-α, and increased the mRNA levels of anti-inflammatory cytokines such as IL-10. Jeschke *et al* (47) found that recombinant HGF reduced burn-related damage to the small intestine. The serum levels of TNF-α, IL-1β and IL-6 were higher in the HGF-treated group than in the control group. However, Jeschke *et al* (47) did not explain why the levels of these cytokines were increased by HGF. Our data indicate that the number of CD4^+^ cells decreased, but the levels of TNF-α, IL-1β and IL-6, as well as those of Th1 and Th2 cytokines such as IL-2, IFN-γ and IL-4, were elevated in the Ad.HGF-treated group. We hypothesize that the reasons for the differences between our findings and those of previous studies may involve differences among mouse strains, our use of intramuscular gene administration mediated by an Ad, and our selection of the early phase of DSS colitis for analysis of inflammation and cytokine expression.

Futamatsu *et al* (48) reported that HGF suppressed T-cell proliferation and IFN-γ production and increased IL-4 and IL-10 secretion from CD4^+^ T cells *in vitro*, and also reduced the severity of experimental autoimmune myocarditis *in vivo* by inducing Th2 cytokines and suppressing apoptosis of cardiomyocytes. Kuroiwa *et al* (49) demonstrated that HGF gene delivery inhibited Th2 immune responses and ameliorated lupus nephritis, autoimmune sialadenitis, and cholangitis in chronic GVHD mice. Another study indicated that treatment with HGF potently suppressed dendritic cell functions such as antigen-presenting capacity, both *in vitro* and *in vivo*, thus downregulating antigen-induced Th1 and Th2 immune responses in a mouse model of allergic airway inflammation (50). HGF has been suggested to suppress airway hyper-responsiveness, inflammation, remodeling, and eosinophil function in asthma (51). Okunishi *et al* (52) reported that HGF suppresses antigen-induced T-cell priming by regulating the functions of dendritic cells through IL-10 downregulation in the antigen-sensitization phase. By contrast, they found that repeated treatment with HGF induced Th2 immune responses with the upregulation of IL-10 by DCs in the chronic inflammation phase of a mouse model of collagen-induced arthritis. Thus, it is clear that HGF induces various immune responses in different disease models. However, further analysis is required to clarify the effects of HGF on the immune system.

In conclusion, we have shown that a single round of intramuscular injections of adenoviral hHGF is sufficient to inhibit apoptosis and reconstitute the epithelium in a mouse model of DSS-induced colitis. Based on these results, this approach shows promise for clinical application in IBD.

## References

[1] Hisamatsu T, Kanai T, Mikami Y, Yoneno K, Matsuoka K, Hibi T (2013). Immune aspects of the pathogenesis of inflammatory bowel disease. Pharmacol Ther.

[2] Bernklev T, Jahnsen J, Aadland E (2004). Health-related quality of life in patients with inflammatory bowel disease five years after the initial diagnosis. Scand J Gastroenterol.

[3] Burger D, Travis S (2011). Conventional medical management of inflammatory bowel disease. Gastroenterology.

[4] Nakamura T, Nawa K, Ichihara A (1984). Partial purification and characterization of hepatocyte growth factor from serum of hepatectomized rats. Biochem Biophys Res Commun.

[5] Russell WE, McGowan JA, Bucher NL (1984). Partial characterization of a hepatocyte growth factor from rat platelets. J Cell Physiol.

[6] Thaler FJ, Michalopoulos GK (1985). Hepatopoietin A: partial characterization and trypsin activation of a hepatocyte growth factor. Cancer Res.

[7] Gohda E, Tsubouchi H, Nakayama H (1986). Human hepatocyte growth factor in plasma from patients with fulminant hepatic failure. Exp Cell Res.

[8] Nakamura T, Nishizawa T, Hagiya M (1989). Molecular cloning and expression of human hepatocyte growth factor. Nature.

[9] Miyazawa K, Tsubouchi H, Naka D (1989). Molecular cloning and sequence analysis of cDNA for human hepatocyte growth factor. Biochem Biophys Res Commun.

[10] Matsumoto K, Nakamura T (1997). Hepatocyte growth factor (HGF) as a tissue organizer for organogenesis and regeneration. Biochem Biophys Res Commun.

[11] Matsuno M, Shiota G, Umeki K, Kawasaki H, Kojo H, Miura K (1997). Clinical evaluation of hepatocyte growth factor in patients with gastrointestinal and pancreatic diseases with special reference to inflammatory bowel disease. Res Commun Mol Pathol Pharmacol.

[12] Kitamura S, Kondo S, Shinomura Y (2000). Expression of hepatocyte growth factor and c-met in ulcerative colitis. Inflamm Res.

[13] Srivastava M, Zurakowski D, Cheifetz P, Leichtner A, Bousvaros A (2001). Elevated serum hepatocyte growth factor in children and young adults with inflammatory bowel disease. J Pediatr Gastroenterol Nutr.

[14] Ortega-Cava CF, Ishihara S, Kawashima K (2002). Hepatocyte growth factor expression in dextran sodium sulfate-induced colitis in rats. Dig Dis Sci.

[15] Dignass AU, Lynch-Devaney K, Podolsky DK (1994). Hepatocyte growth factor/scatter factor modulates intestinal epithelial cell proliferation and migration. Biochem Biophys Res Commun.

[16] Numata M, Ido A, Moriuchi A (2005). Hepatocyte growth factor facilitates the repair of large colonic ulcers in 2,4,6-trinitrobenzene sulfonic acid-induced colitis in rats. Inflamm Bowel Dis.

[17] Tahara Y, Ido A, Yamamoto S (2003). Hepatocyte growth factor facilitates colonic mucosal repair in experimental ulcerative colitis in rats. J Pharmacol Exp Ther.

[18] Ohda Y, Hori K, Tomita T (2005). Effects of hepatocyte growth factor on rat inflammatory bowel disease models. Dig Dis Sci.

[19] Setoyama H, Ido A, Numata M (2011). Repeated enemas with hepatocyte growth factor selectively stimulate epithelial cell proliferation of injured mucosa in rats with experimental colitis. Life Sci.

[20] Arthur LG, Schwartz MZ, Kuenzler KA, Birbe R (2004). Hepatocyte growth factor treatment ameliorates diarrhea and bowel inflammation in a rat model of inflammatory bowel disease. J Pediatr Surg.

[21] Mukoyama T, Kanbe T, Murai R (2005). Therapeutic effect of adenoviral-mediated hepatocyte growth factor gene administration on TNBS-induced colitis in mice. Biochem Biophys Res Commun.

[22] Hanawa T, Suzuki K, Kawauchi Y (2006). Attenuation of mouse acute colitis by naked hepatocyte growth factor gene transfer into the liver. J Gene Med.

[23] Kanbe T, Murai R, Mukoyama T (2006). Naked gene therapy of hepatocyte growth factor for dextran sulfate sodium-induced colitis in mice. Biochem Biophys Res Commun.

[24] Kanayama M, Takahara T, Yata Y (2007). Hepatocyte growth factor promotes colonic epithelial regeneration via Akt signaling. Am J Physiol Gastrointest Liver Physiol.

[25] Li Y, Takemura G, Kosai K (2003). Postinfarction treatment with an adenoviral vector expressing hepatocyte growth factor relieves chronic left ventricular remodeling and dysfunction in mice. Circulation.

[26] Chen SH, Chen XH, Wang Y (1995). Combination gene therapy for liver metastasis of colon carcinoma in vivo. Proc Natl Acad Sci USA.

[27] Takahashi T, Kawai T, Ushikoshi H (2006). Identification and isolation of embryonic stem cell-derived target cells by adenoviral conditional targeting. Mol Ther.

[28] Okabe Y, Kusaga A, Takahashi T (2010). Neural development of methyl-CpG-binding protein 2 null embryonic stem cells: a system for studying Rett syndrome. Brain Res.

[29] Horikawa Y, Wang Y, Nagano S (2011). Assessment of an altered E1B promoter on the specificity and potency of triple-regulated conditionally replicating adenoviruses: implications for the generation of ideal m-CRAs. Cancer Gene Ther.

[30] Okayasu I, Hatakeyama S, Yamada M, Ohkusa T, Inagaki Y, Nakaya R (1990). A novel method in the induction of reliable experimental acute and chronic ulcerative colitis in mice. Gastroenterology.

[31] Tomoyose M, Mitsuyama K, Ishida H, Toyonaga A, Tanikawa K (1998). Role of interleukin-10 in a murine model of dextran sulfate sodium-induced colitis. Scand J Gastroenterol.

[32] Kanauchi O, Nakamura T, Agata K, Mitsuyama K, Iwanaga T (1998). Effects of germinated barley foodstuff on dextran sulfate sodium-induced colitis in rats. J Gastroenterol.

[33] Yuge K, Takahashi T, Nagano S (2005). Adenoviral gene transduction of hepatocyte growth factor elicits inhibitory effects for hepatoma. Int J Oncol.

[34] Kamisasanuki T, Tokushige S, Terasaki H (2011). Targeting CD9 produces stimulus-independent antiangiogenic effects predominantly in activated endothelial cells during angiogenesis: a novel antiangiogenic therapy. Biochem Biophys Res Commun.

[35] Murthy SN, Cooper HS, Shim H, Shah RS, Ibrahim SA, Sedergran DJ (1993). Treatment of dextran sulfate sodium-induced murine colitis by intracolonic cyclosporin. Dig Dis Sci.

[36] Li Y, Takemura G, Kosai K (2004). Critical roles for the Fas/Fas ligand system in postinfarction ventricular remodeling and heart failure. Circ Res.

[37] Iwamoto M, Koji T, Makiyama K, Kobayashi N, Nakane PK (1996). Apoptosis of crypt epithelial cells in ulcerative colitis. J Pathol.

[38] Sträter J, Wellisch I, Riedl S (1997). CD95 (APO-1/Fas)-mediated apoptosis in colon epithelial cells: a possible role in ulcerative colitis. Gastroenterology.

[39] Rogler G, Andus T (1998). Cytokines in inflammatory bowel disease. World J Surg.

[40] Egger B, Bajaj-Elliott M, MacDonald TT, Inglin R, Eysselein VE, Büchler MW (2000). Characterisation of acute murine dextran sodium sulphate colitis: cytokine profile and dose dependency. Digestion.

[41] Tomanin R, Scarpa M (2004). Why do we need new gene therapy viral vectors? Characteristics, limitations and future perspectives of viral vector transduction. Curr Gene Ther.

[42] Boulaiz H, Marchal JA, Prados J, Melguizo C, Aránega A (2005). Non-viral and viral vectors for gene therapy. Cell Mol Biol (Noisy-le-grand).

[43] Oh K, Iimuro Y, Takeuchi M (2005). Ameliorating effect of hepatocyte growth factor on inflammatory bowel disease in a murine model. Am J Physiol Gastrointest Liver Physiol.

[44] Shiota G, Kawasaki H, Nakamura T, Schmidt EV (1995). Characterization of double transgenic mice expressing hepatocye growth factor and transforming growth factor alpha. Res Commun Mol Pathol Pharmacol.

[45] Sakata H, Takayama H, Sharp R, Rubin JS, Merlino G, LaRochelle WJ (1996). Hepatocyte growth factor/scatter factor overexpression induces growth, abnormal development, and tumor formation in transgenic mouse livers. Cell Growth Differ.

[46] Chen P, Kovesdi I, Bruder JT (2000). Effective repeat administration with adenovirus vectors to the muscle. Gene Ther.

[47] Jeschke MG, Bolder U, Finnerty CC (2005). The effect of hepatocyte growth factor on gut mucosal apoptosis and proliferation, and cellular mediators after severe trauma. Surgery.

[48] Futamatsu H, Suzuki J, Mizuno S (2005). Hepatocyte growth factor ameliorates the progression of experimental autoimmune myocarditis: a potential role for induction of T helper 2 cytokines. Circ Res.

[49] Kuroiwa T, Iwasaki T, Imado T, Sekiguchi M, Fujimoto J, Sano H (2006). Hepatocyte growth factor prevents lupus nephritis in a murine lupus model of chronic graft-versus-host disease. Arthritis Res Ther.

[50] Okunishi K, Dohi M, Nakagome K (2005). A novel role of hepatocyte growth factor as an immune regulator through suppressing dendritic cell function. J Immunol.

[51] Ito W, Takeda M, Tanabe M (2008). Anti-allergic inflammatory effects of hepatocyte growth factor. Int Arch Allergy Immunol.

[52] Okunishi K, Dohi M, Fujio K (2007). Hepatocyte growth factor significantly suppresses collagen-induced arthritis in mice. J Immunol.

